# Physical Activity, Energy Expenditure, Nutritional Habits, Quality of Sleep and Stress Levels in Shift-Working Health Care Personnel

**DOI:** 10.1371/journal.pone.0169983

**Published:** 2017-01-12

**Authors:** Frederick Charles Roskoden, Janine Krüger, Lena Johanna Vogt, Simone Gärtner, Hans Joachim Hannich, Antje Steveling, Markus M. Lerch, Ali A. Aghdassi

**Affiliations:** 1 Department of Medicine A, University Medicine Greifswald, Greifswald, Germany; 2 Institute of Medical Psychology, University Medicine Greifswald, Greifswald, Germany; Old Dominion University, UNITED STATES

## Abstract

**Background:**

Among health care personnel working regular hours or rotating shifts can affect parameters of general health and nutrition. We have investigated physical activity, sleep quality, metabolic activity and stress levels in health care workers from both groups.

**Methods:**

We prospectively recruited 46 volunteer participants from the workforce of a University Medical Department of which 23 worked in rotating shifts (all nursing) and 21 non-shift regular hours (10 nursing, 13 clerical staff). All were investigated over 7 days by multisensory accelerometer (SenseWear Bodymedia® armband) and kept a detailed food diary. Physical activity and resting energy expenditure (REE) were measured in metabolic equivalents of task (METs). Quality of sleep was assessed as Pittsburgh Sleeping Quality Index and stress load using the Trier Inventory for Chronic Stress questionnaire (TICS).

**Results:**

No significant differences were found for overall physical activity, steps per minute, time of exceeding the 3 METs level or sleep quality. A significant difference for physical activity during working hours was found between shift-workers vs. non-shift-workers (p<0.01) and for shift-working nurses (median = 2.1 METs SE = 0.1) vs. non-shift-working clerical personnel (median = 1.5 METs SE = 0.07, p<0.05). Non-shift-working nurses had a significantly lower REE than the other groups (p<0.05). The proportion of fat in the diet was significantly higher (p<0.05) in the office worker group (median = 42% SE = 1.2) whereas shift-working nurses consumed significantly more carbohydrates (median = 46% SE = 1.4) than clerical staff (median = 41% SE = 1.7). Stress assessment by TICS confirmed a significantly higher level of social overload in the shift working group (p<0.05).

**Conclusion:**

In this prospective cohort study shift-working had no influence on overall physical activity. Lower physical activity during working hours appears to be compensated for during off-hours. Differences in nutritional habits and stress load warrant larger scale trials to determine the effect on implicit health-associated conditions.

## Introduction

The effects on health in individuals being on different working schedules (daytime, night-time or rotating) are a widely discussed topic and cover not only metabolic syndrome and its risk factors like obesity [1]. Cardiovascular as well as gastrointestinal disorders and cancer were identified in several review papers containing smaller national studies [2,3,4,5].

In a generic large scaled study Japanese shift-workers were found to have a higher prevalence of gastric ulcera then day time workers (2.38 vs 1.03%) [6].

Moreover a statistical analysis of a large Scandinavian working cohort comparing day-time workers and shift workers showed a. significantly elevated body-mass-index (BMI) >30 kg∙m^-2^ for female workers of all age groups and for male workers in the third, fourth and fifth decade of life. Significantly elevated triglyceride levels above 1.7% were observed for female shift workers in their fourth and sixth decade of life [4]. Though risk clusters are found in shift-working cohorts.

Employees in the health care industry are frequently facing different working patterns that might influence their health and both physical and psychological wellbeing [1,7].

A prospective 4-follow-up study in male and female nurses showed a significantly elevated relative risk of 5.0 (95% confidence-interval 2.1–14.6) for de novo developing a component of metabolic syndrome in night-shift workers compared to only day-shift workers [1].

High prevalence of poor sleeping quality measured by a questionnaire was found examining resident physicians doing 24 hours of shift work [7].

Epidemiological studies reported that declining physical activity (PA) or an increasing sedentary activity is associated with higher risks for health such as poor insulin sensitivity [8]. The substitution of 30 minutes sedentary time with moderate to vigorous physical activity (MVPA) was found associated with a 9.7% higher homeostasis model assessment of insulin sensitivity [9].

A lack of moderate PA is considered to be responsible for metabolic disorders such as metabolic syndrome. A decrease of 68%–81% for risks of less likely having abdominal obesity, hypertriglyceridemia, and low HDL cholesterol levels was seen in a study cohort using a SenseWear armband and spending more than 60 minutes per day with MVPA compared to those spending less than 30 minutes per day of MVPA[10].

Sedentariness as a possible predictor for developing metabolic syndrome was found with a significantly higher hazard ratio of 5.10 (95% confidence interval 2.15 to 12.11; p<0.001) in night shift workers compared to day time workers with a hazard ratio of 2.92 (95% confidence interval 1.64 to 5.18; p = 0.017) [1].

Other data suggest a relative risk of 1.79 (95% confidence interval 1.06–3.01) for breast cancer in rotating night-shift workers compared with non-shifting workers [11] That risk as well as the risk for cardiovascular disease in persons on night work might be associated with suppressed nocturnal melatonin levels, supporting the ‘melatonin hypothesis’ [12].

Less bouts of moderate to MVPA were observed in night and evening shift workers compared to day shift workers. A rotating shift schedule was associated with decreased sedentary but more light physical activity in the National Health and Nutrition Examination Survey [13].

Health care personnel, especially nurses, are a well investigated population for health studies. Since 1976, the “Nurses’ Health Study” examines nurses for health-risks and lifestyle parameters like e.g. smoking and nutrition [14] and is currently recruiting its third cohort.

Investigations of individual physical activity are becoming more attractive due to the increasing availability of easy to handle fitness tracking devices. Unfortunately, due to the inconsistent design of studies and heterogeneous results a generalization of observations is not possible. It appears probable that circadian rhythm and sleep is negatively affected by shift work [3], which is expressed by subjective sleepiness and short sleeping periods between shifts [15].

In addition to physical activity and sleeping patterns eating habits have been investigated in shift workers and evaluated for their impact for the risk of metabolic syndrome [4,5]. A recent study revealed no differences in nutritional behaviour between regular and alternating shift workers that might be explained by equal health beliefs in both groups [16].

The so called healthy worker effect proposes that the apparently better health of workers contrasts always with the general population. Therefore workers need to be compared to appropriate controls [17].The individualized and multifactorial objective of the present study draws on the recent approach of the biopsychosocial model of GL Engel [18]. This model does not take into account solely the biological impacts of one individual’s health and illness but also involves psychological aspects like mental disposition and personality and social environment such as education, work, family etc. Understanding and analysing characteristics of health care staff and shift worker, which increase risk for illness, our study should not only measure one biological parameter such as physical activity. Measuring chronic stress and sleep quality are aimed to cover psychological aspects. The social aspects of work should be represented in the compared subgroups. The influence of shift-work as well as the type of work are supposed to be integrated into analysis. The need for an outlying (no shift-work, no health-care work) but likely comparable subgroup was acknowledged.

In health care many employees have to work either in a daytime or shift-working schedule and this group has not been extensively characterized regarding their physical activity, sleeping rhythm and nutritional behaviour. Our aim was to clarify the impact of shift working in these groups.

## Materials and Methods

**Fig 1:**
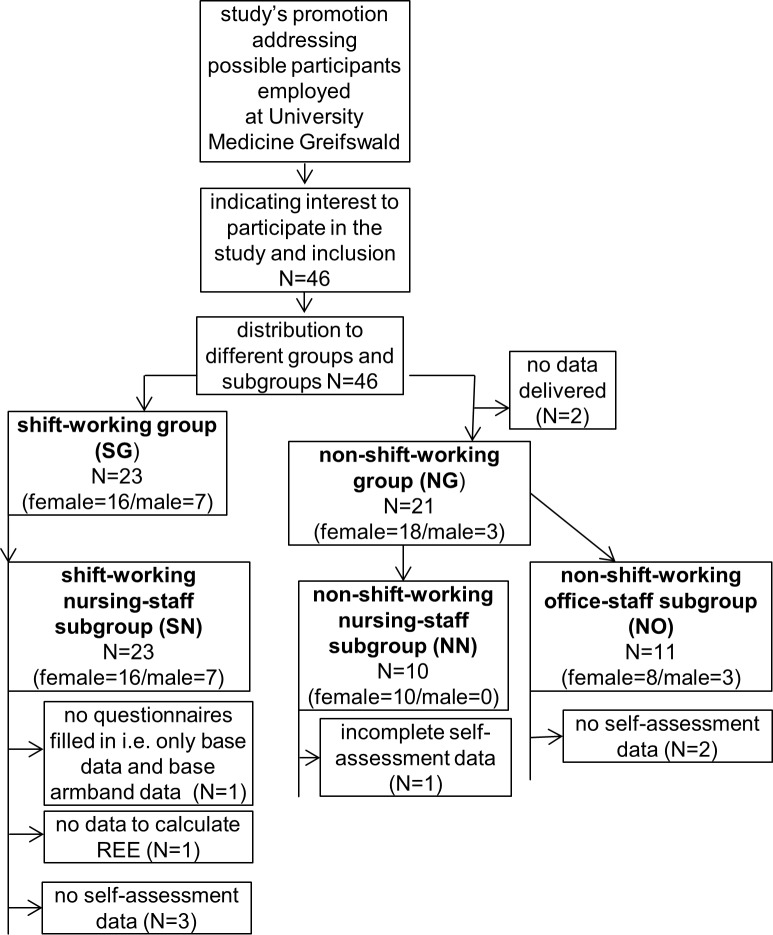
Study’s flowchart for group distribution, group sizes as well as amount of missing data. The shift-working group (SG) only consisted of nursing staff (male and female) from different wards of University Medicine Greifswald. They rotated through day-, evening- and night-shifts. The non-shift-working group (NG) included nurses that worked in outpatient departments, diagnostic units or solely on day-shift on the wards (NN), as well as office administrative staff (NO).

The study population consisted of employees of University Medicine Greifswald, a tertiary care medical centre in Western Pomerania, Germany. The study was approved by the Local Ethics Committee of the University of Greifswald (‘Ethikkommission Universitätsmedizin Greifswald’, Felix-Hausdorff-Str. 3, 17487 Greifswald, Germany) on July 2013. All volunteers signed informed consent. Volunteers were recruited among the nursing and administration staff between 2013 and 2014. All participants were aged above 18 years and had an unremarkable medical history except for e.g. occasional back pain or arterial hypertension. No financial compensation was offered, but all participants were offered to receive their individual results after termination of the study. Participants were processed as outlined in Fig 1.

### Anthropometrics

Age, weight, height, waist and hip circumferences, left- or right-handedness, nicotine consumption, blood pressure and heart rate were recorded at the beginning of the study. Besides BMI waist-to-hip-ratio (WHR) was calculated. The recent Expert Consultation of the World Health Organization acknowledges the use of WHR and the waist circumference solely as a parameter for abdominal obesity. Appropriate cut-off points for different ethnicities and schemes for scaling are still controversial. Although World Health Organization’s possible cut-offs of about ≥0.90 cm/cm for men and ≥0.85 cm/cm for women are suggested to indicate a ‘substantially increased risk of metabolic complications’ [19].

The WHR considers the distribution of body fat and has been suggested to represent a better predictor for the occurrence of cardiovascular diseases and their risk factors than BMI e.g. better indicating dyslipidaemia or hypertension [20].

### Bioelectrical impedance analysis

Body composition was measured using bioelectrical impedance analysis (BIA, Nutriguard-M and the included NutriPlus® software by Data Input GmbH Pöcking, Germany). This non-invasive method is frequently used in both clinical routine as well as in trials for assessment of dietary and physical conditions. A special focus was laid on the assessment of body fat, body water and body cellular mass (BCM). For better interpersonal comparability the ratio of BCM and extracellular mass (ECM), BCM/ECM index, and the phase angle as a parameter for muscular cell vitality were determined.

### SensewearPro armband accelerometry and physical activity

The physical activity was measured (actigraphy) by the SensewearPro3 armband (SWA, Bodymedia Inc. Pittsburgh, PA, USA). This device is a multisensory research-grade accelerometer worn at the upper arm (regio brachialis posterior), registering longitudinal and transversal acceleration, number of steps, rate of heat-dissipation, galvanic skin response, body and ambient temperature. Data were recorded constantly every minute. SWA is a valid and reliable tool for total energy expenditure and resting energy expenditure (REE) when compared to the gold standard indirect calorimetry [21].

Physical activity was quantified as metabolic equivalents of task (MET). The equivalence of 1 MET is defined by the turnover of 3.5 ml oxygen per kg body weight per minute. In other words, it can be defined as the turnover of 1 kcal (= 4.2 kJ) per kg body weight per minute. In normo-metabolic individuals 1 MET represents the activity level of 1 minute of quiet sitting [22].The activity-levels were defined as light activity (<3.0 METs), moderate activity (≥3.0 to <6 METs), vigorous activity (≥6 to <9.0 METs) and highly vigorous activity (≥9.0 METs) as previously reported [22].

The American College of Sports Medicine and the American Heart Association recommends 30 minutes (i.e. 2.1% of an entire day’s time) of MVPA preferably in 10 min bouts, per day for healthy adults [23]. In definition of METs all activities above the 3 METs threshold would be in compliance with this recommendation.

All subjects were asked to wear the armband continuously for 1 week to cover five working days and an equal distribution of different shift-types in the SG subgroup although this was not always feasible. In addition, all subjects had to record the start and the end of their working hours, sleeping and resting hours, and optionally, their leisure time activities in a log handed to them at the beginning of the study.

All processed parameters, including the individual METs (through the temporal dimension), were standardized to the individual’s effective wearing time to ensure general comparability. The value of subjects’ individual METs was calculated by SWA’s attached software, through a special algorithm.

Furthermore, we analysed the percentage of time exceeding the 3 METs level, steps per minute and the percentage of time in all activity categories (see above), which were recorded by the armband. Through the subjects log recording we could extract all these parameters for the entire working period.

### Metabolism and REE

Owing the fact that 1 MET represents the activity of quiet sitting in the presence of a normal metabolism; METs in resting subjects can represent the individual REE. REE was calculated on the basis of the MET-level of 1 hour activity during the early sleeping phase that was retrieved from the subjects’ log. The mean REE (calculated in METs) of all measurements was calculated for each subject.

Depending on the individual body composition and diet a REE of <0.9 METs defines hypometabolism, between 0.9 and 1.1 normometabolism and >1.1 hypermetabolism.

### Sleep quality and efficiency

The SensewearPro armband provides the opportunity to evaluate the sleeping habits of its wearer. Laying and sleeping times were registered and sleeping hours were identified according to the diary. The mean ratio of all hours of lying and all hours of sleeping represents the individual sleeping efficiency (in percent). Actigraphy is, in general, regarded as valid to assess sleeping quality [24], but certain limitations in the detection of sleep and wake times have been discussed [25]. We therefore used as additional instrument, the Pittsburgh Sleeping Quality Index (PSQI). A 19-item based self-reporting questionnaire covering subjective sleep quality, latency and duration, habitual sleep efficiency, sleep disturbances, use of sleeping medication, and daytime dysfunction [26]. A PSQI score >5 indicated poor sleeping quality.

### Nutritional habits

All participants were asked to keep a food diary for 7 days. All non-nutritional beverages (pure water and unsweetened tea) were neglected for the sake of compliance and practicability. The data was analysed using the OptiDiet© software (GOE GmbH Linden, Germany). Intake of macronutrients and micronutrients were quantified using an integrated database of the software. The average percentage intake of fat, carbohydrates, proteins was determined by the software. Intake sugar (sucrose) as a subset of carbohydrate as analysed especially again

### Quantification and differentiation of stress load

The Trier Inventory for Assessment of Chronic Stress (TICS) is a validated 57-item questionnaire [27], which estimates individual chronic stress loads during the preceding 3 months. Items can be categorized in 10 groups: work overload, social overload, pressure to perform, work discontent, excessive demands from work, lack of social recognition, social tensions, social isolation, chronic worrying and stress screening scale, a global indicator for stress load. All results were calculated by an age-adjusted T-value and found positive when this value ±95%-confidence interval exceeded the mean T-value of 50. The study used a one-sided approach of TICS utilisation and neglected significant deceeding values. Comparisons between the groups were done in categories of positive and negative outcome. Positive values indicate higher perception of the respective stress type.

### Statistics

Statistical analysis and graphical editing were done using SAS 9.3 software (SAS Institute Cary, NC, USA) and Sigma Plot 11.0 (Systat Software Inc. San Jose, CA, USA).

If not otherwise indicated the median and standard error (SE) is presented for descriptive statistics, to ensure robust statistics. Nevertheless for general data the mean-value is also delivered (see General Data).

For categorical variables Fisher’s Exact Test was performed. In case of continuous variables ANOVA was used. ANOVA’s assumptions of normal distribution were tested with the Shapiro-Wilk test and the requirement of homogeneity of variances was tested with Levene’s test and the more robust Brown-Forsythe test.

When ANOVA assumptions were violated, the non-parametric Wilcoxon–Mann–Whitney test was used to compare two samples (group comparison) and Kruskal-Wallis test to compare three samples (subgroup analysis). Since continuity correction for Wilcoxon–Mann–Whitney test can be neglected only for larger sample sizes or absence of ties, a correction of 0.5 was kept.

The post-hoc analysis for the three subgroups could be done for ANOVA agreements performing Tukey’s test, controlling the type I experimentwise error rate. For the non-parametric test and categorical variables the subgroup analysis was done using Bonferroni correction for multiple testing.

Unless otherwise indicated a p-value of <0.05 was considered to be significant. For the subgroup analysis the levels of significance refer to the first step of testing for difference and not to the post-hoc analysis.

## Results

### General data

The study population categorized in shift working and non-shift working employees and their subgroups are characterized in Table 1. Non-shift workers were significantly (p<0.001) older than individuals working on a shift schedule showing a difference of about 10 years (41.6±2.0 vs. 31±1.9 years). Among non-shift workers, nurses were older than office staff. Study participation of males was lower as only 10 out of 44 individuals were men; however this difference was not significant.

**Table 1:** Main characteristics of the study population and respective subpopulations.

(sub)population characteristics	shift-working group *RESPECTIVELY* shift-working nursing-staff subgroup (N = 23)	non-shift-working group (N = 21)	non-shift-working nursing-staff subgroup (N = 10)	non-shift-working office-staff subgroup (N = 11)
Age (yr)	31±1.9 (27)	41.62±2.0 (43)***	46.7±1.5 (47) *	37±3.0 (33)
Height (cm)	172±2.4 (170)	168±1.3 (167)	167.5±1.3 (167.5)	169±2.1 (167)
Weight (kg)	76.35±4.2 (72)	72±3.3 (72)	75.7±5.1 (74)	68.6±4.2 (65)
BMI in (kg·m^-2^)	25.7±1.3 (23.8)	25.4±1.1 (24.2)	27.1±2 (26.0)	23.9±1.07 (23.9)
Waist-to-hip-ratio	0.86±0.01(0.86)	0.84±0.02 (0.85)	0.84±0.02 (0.86)	0.84±0.03 (0.85)
Armband-wearing-rate (%)	96.5±1.00 (98.4)	93.0±2.4 (97.6)	89.6±4.8 (97.5)	96.1±1.3 (97.8)
Packyears (in years)	4.3±1.2 (3)*	1.8±0.8 (0)	2.5±1.5 (0)	1.18±0.6 (0)
Phase angle (°)	6.08±0.16 (6.00)	5.93±0.13 (6.00)	5.92±0.16 (5.70)	5.95±0.22 (6.00)
Body cellular mass (%)	52.1±0.8(52)	51.5±0.7 (51.7)	51.5±0.8 (50.5)	51.48±1.03 (51.8)
Body fat (%)	26.0±2.24 (25.2)	27.09±1.57 (24.6)	30.5±2.7 (33.5)	24.0±1.3 (23.6)

values are means ± SE additionally; median-values are in brackets.

*p<0.05

***p<0.001

BMI and WHR did not show significant differences within the groups and between subgroups. The median BMI and the median WHR in all subpopulation was within the range of healthy individuals. For BMI a tendency towards overweight was found for all subgroups, whereas the NN subgroup has the highest median BMI of all subgroups.

Smoking habits estimated by the number of pack years indicated that individuals on a shift schedule smoked significantly more than employees working during daytime only (4.3±1.2 vs. 1.8±0.8 pack years).

A potential bias for the percental SWA wearing rate in the main groups could be assumed, apartly beholding a very conservative view at the two tailed p-value (p = 0.0926) and the one-tailed p-value (p = 0.0463).

### Physical activity and metabolism

**Fig 2:**
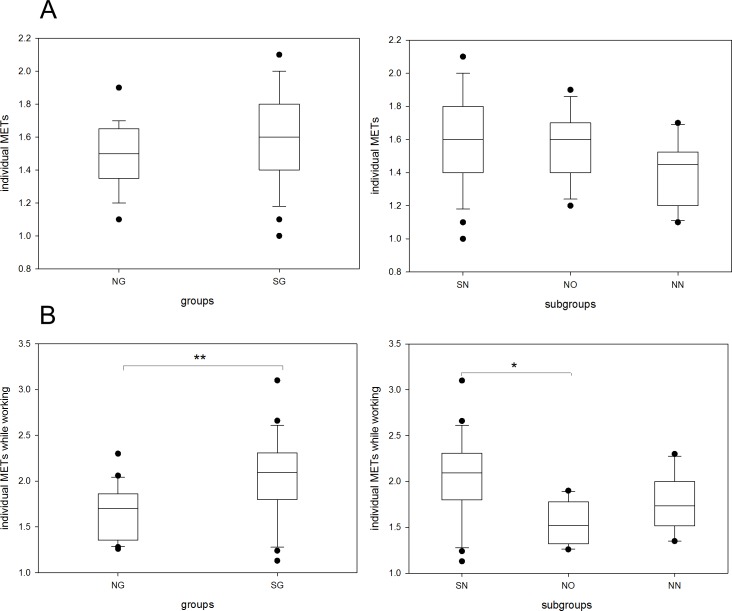
Individual physical activity in metabolic equivalents of task (METs) in shift and non-shift workers and their subgroups. **A: induvial physical activity for overall time in METs. B: individual physical activity in METs during the working time** The boxes cover the first quartile on the bottom and the third quartile on the top. Whiskers reach from the minimum to the maximum value excluding outliers (illustrated by dots). Shift-working group and shift-working nursing-staff subgroup cover identical cohorts. Working periods were identified using the test subject’s activity journal and analysed separately. The value of subjects’ individual METs was calculated by SWA’s attached software, through a special algorithm. NG, non-shift-working group; SG, shift-working group; SN, shift-working nursing-staff subgroup; NO, non-shift-working office-staff subgroup; NN, non-shift-working nursing-staff subgroup. *p<0.05, **p<0.01.

Overall average activity in METs was not significantly different between the shift- and non-shift working group and ranged at around 1.6 METs (Fig 2A). Regarding the subgroups, no statistically significant differences were detected either. There was a tendency towards less physical activity in the non-shift working nursing staff subgroup compared to employees from the office staff. No significant differences were found for different activity levels in METs for overall wearing time.

However, when analysing the median physical activity during business hours shift-working individuals were significantly more active than non-shift workers (2.1 vs. 1.7 METs, p<0.01). In addition, there was a significant difference between shift-working nurses and (non-shift working) office workers (2.095 vs. 1.52 METs, p<0,05) (Fig 2B).

There was also a significant difference in the percentage of time exceeding more than 3.0 METs per minute of wearing during working phases for shift-workers and non-shift workers.

We tend to define the parameter “percentage of time exceeding more than 3.0 METs per minute of wearing” for overall time of as analogous to the common MVPA, in which 30 minutes of MVPA (i.e. 2.1% of an entire day’s time) [23] are recommended. In this case all subgroups would accomplish the recommendation for sufficient MVPA. The median values for SN is 10.8% (SE = 1.23), for NN = 8.6% is (SE = 1.25), for NO is 10.5% (SE = 1.4).

In working phases we also found significant differences between shift- and non-shift-workers regarding the percentage of time reaching levels of low (<3.0 METs) and medium activity (3.0–6.0 METs).

**Fig 3:**
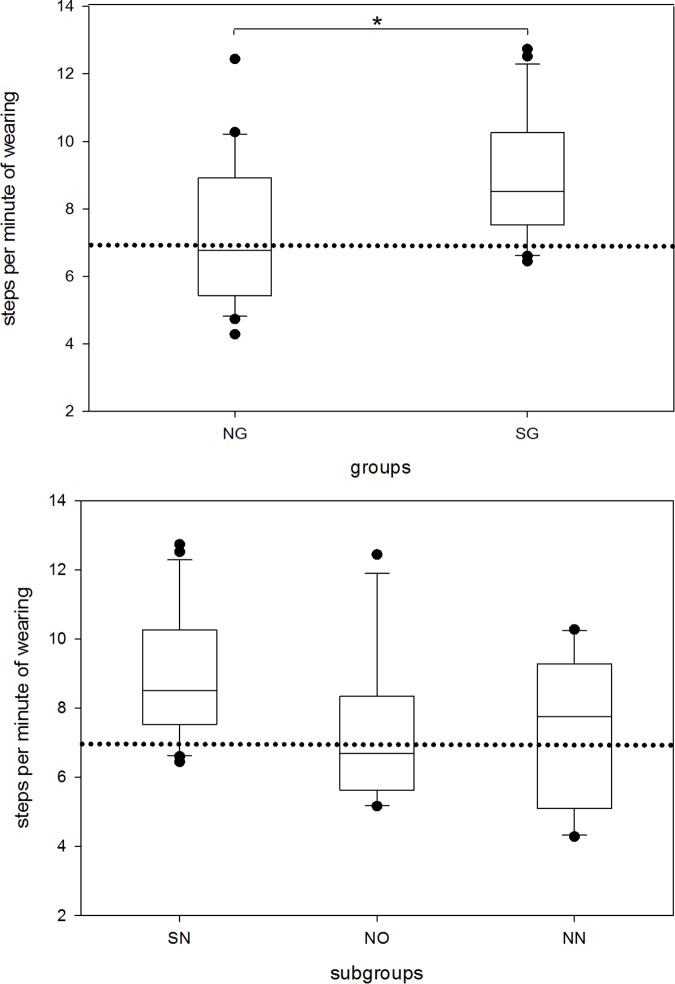
Overall steps per minute in non-shift and shift working group and subgroups. The boxes cover the first quartile on the bottom and the third quartile on the top. Whiskers reach from the minimum to the maximum value excluding outliers (illustrated by dots). Shift-working group and shift-working nursing-staff subgroup cover identical cohorts. Steps per minute were calculated based on the ratio of overall steps and overall armband wearing time in minutes. Dotted line represents the 10000 steps per day boundary (i.e. 6.94 steps·min^-1^). NG, non-shift-working group; SG, shift-working group; SN, shift-working nursing-staff subgroup; NO, non-shift-working office-staff subgroup; NN, non-shift-working nursing-staff subgroup *p<0.05.

We then focussed on the number of steps (per minute of wearing) as a health-related activity parameter (Fig 3). There was a significant difference between the shift-working and the non-shift-working group (p = 0.012). Employees on shift rotation walked relatively more than individuals on non-shift work over the whole time of wearing. Significance was also found in a subgroup analysis, but did not withstand a controlled pairwise ANOVA post-hoc analysis using the Tukey test.

When comparing individual METs (Fig 2A) and steps per minute (Fig 3) we found a significant difference(p<0.001) but poor correlation (spearman‘s rho = 0.559) of those variables, suggesting that other activities rather than walking might be responsible for accomplishing the total metabolic equivalents of task in our investigated population.

During the working phases the difference in number of steps per minute was greatest between the NG and SG cohort (p<0.0001, data not shown) and there were significant pairwise differences between all subgroups.

Correlation of METs at work and steps per minute while working was significantly different (p<0.05) and showed strong correlation (Spearman‘s rho = 0.763) (Figs 2B and 3). During work the step-rate depicts the metabolic equivalents of task very precisely.

**Fig 4:**
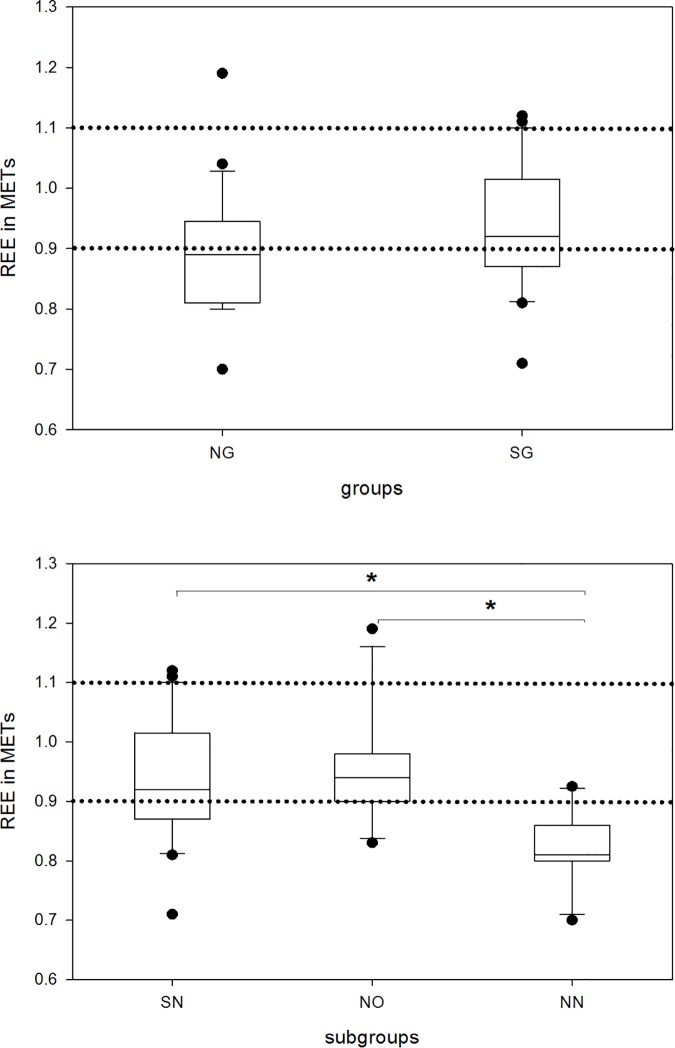
Boxplot of Resting Energy Expenditure (REE) in METS by groups and subgroups. The boxes cover the first quartile on the bottom and the third quartile on the top. Whiskers reach from the minimum to the maximum value excluding outliers (illustrated by dots). Shift-working group and shift-working nursing-staff subgroup cover identical cohorts. The dotted lines bounds the limits for hypermetabolic state (REE>1.1 METs) and hypometabolic state (REE<0.9 METs). NG, non-shift-working group; SG, shift-working group; SN, shift-working nursing-staff subgroup; NO, non-shift-working office-staff subgroup; NN, non-shift-working nursing-staff subgroup *p<0.05

No significant differences in REE were found in the SG and NG groups (Fig 4). This is not remarkable because the main anthropometric characteristics including results of body impedance analysis (Table 1) were also not different. BCM is a predictor, independent of age and gender for REE as it represents metabolically active tissue [28]. Nevertheless, subgroup analysis revealed a significant difference between shift working and non-shift working nurses as well as office workers. Post-hoc analysis showed that NN has a much lower REE compared to the other two subgroups (SN and NO).

Owing to the fact that the REE for woman and man is different, the difference stays significant when only female study subjects are compared. The drawn barriers for different types of metabolism also indicated that non-shift workers, especially NN, are in the majority hypometabolic.

### Sleeping quality

**Fig 5:**
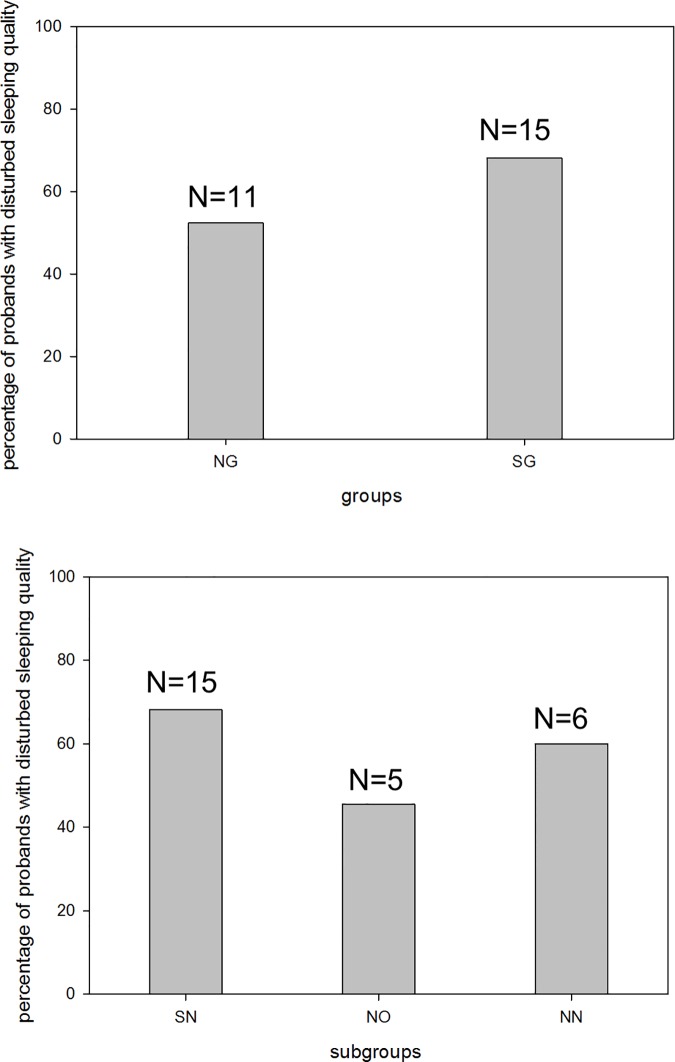
Percentage of employees with disturbed sleeping quality due to Pittsburgh-Sleeping-Quality-Index (PSQI). All test subjects with PSQI-score>5 in groups and subgroups were labelled as having a disturbed sleeping quality. NG, non-shift-working group; SG, shift-working group; SN, shift-working nursing-staff subgroup; NO, non-shift-working office-staff subgroup; NN, non-shift-working nursing-staff subgroup.

Subjective examination of disturbed sleeping quality was assessed by PSQI and showed no significant differences in the group- and subgroup-analyses (Fig 5). Individuals on a shift-work schedule more often reported an impaired sleep quality. Among non-shift workers disturbed sleeping quality was more frequent in non-shift-working nurses than in office staff. Comparison of PSQI results with sleeping efficiency by the SWA-assisted method also showed no significant differences between groups and subgroups.

### Chronic stress

**Fig 6:**
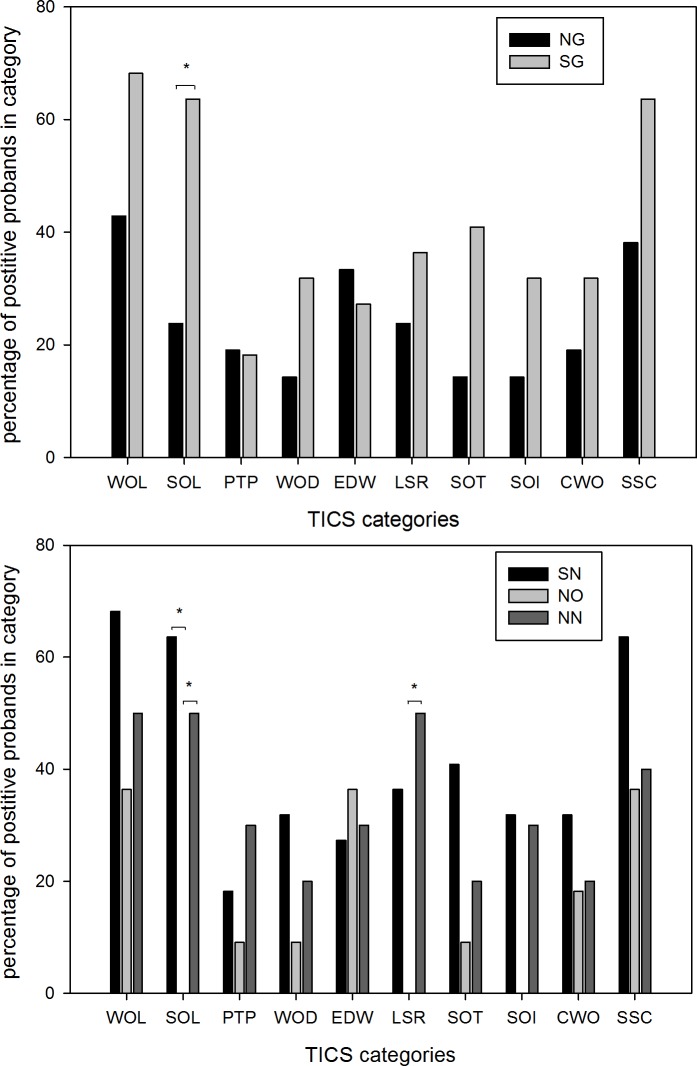
Percentage of different loads in stress categories according to TICS in groups and subgroups. (WOL) work overload, (SOL) social overload, (PTP) pressure to perform, (WOD) work discontent, (EDW) excessive demands from work, (LSR) lack of social recognition, (SOT) social tensions, (SOI) social isolation, (CWO) chronic worrying, (SSC) stress screening scale. NG, non-shift-working group; SG, shift-working group; SN, shift-working nursing-staff subgroup; NO, non-shift-working office-staff subgroup; NN, non-shift-working nursing-staff subgroup *p<0.05.

TICS was used to make a comparison between stress affection in different groups. Fig 6 shows the proportion of study participants that were rated “positive” for each of the ten stress items. Social overload was significantly higher in shift-workers. This observation is based on highly significant differences between the three subgroups. Post-hoc analysis showed that social overload of SN and NN cohorts were strongly increased compared to the NO subgroup. Differences were highest when comparing shift-working nurses with office workers (p<0.001).

TICS defines social overload by a high degree of personal interaction and extended responsibility for other people.

Shift-working employees more frequently complained of work overload). Their stress screening scale, an indicator for global stress load, tended to be higher but not significantly so.

Among non-shift workers a significantly higher lack of social recognition was reported from non-shift-working nurses than from office staff. Comparison of shift-working nurses and clerical staff failed to be significant after Bonferroni correction. According to TICS, lack of social recognition is defined as a mismatch between individual commitment and the expected social gratification.

It should be noted that anybody of the office employees reported on social overload, lack of social recognition or social isolation. Differences were only significant for the first two items before adapting the Bonferroni correction.

### Nutritional issues

**Fig 7:**
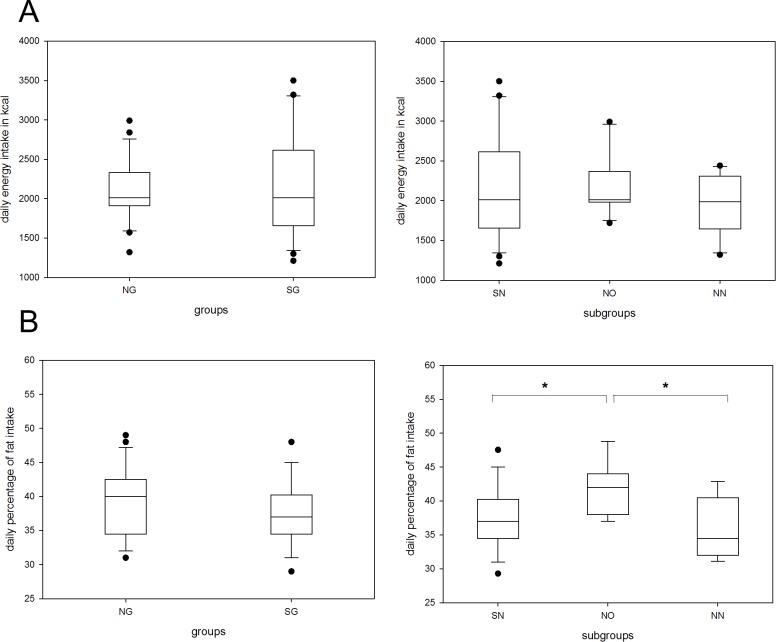
**Daily energy (A) and fat intake (B) by groups and subgroups.** Energy intake is calculated in kcal (A), fat intake (B) as illustrated in percent. The boxes cover the first quartile on the bottom and the third quartile on the top. Whiskers reach from the minimum to the maximum value excluding outliers (illustrated by dots). Shift-working group and shift-working nursing-staff subgroup cover identical cohorts. NG, non-shift-working group; SG, shift-working group; SN, shift-working nursing-staff subgroup; NO, non-shift-working office-staff subgroup; NN, non-shift-working nursing-staff subgroup *p<0.05.

Total energy intake (in kcal) was similar between the shift and non-shift working group as well as in subgroup analysis as illustrated in Fig 7. There was a great variation of energy consumption within the SG and SN group (Fig 7A). Likewise no significant differences were found for percentage of protein ingestion (data not shown).

**Fig 8:**
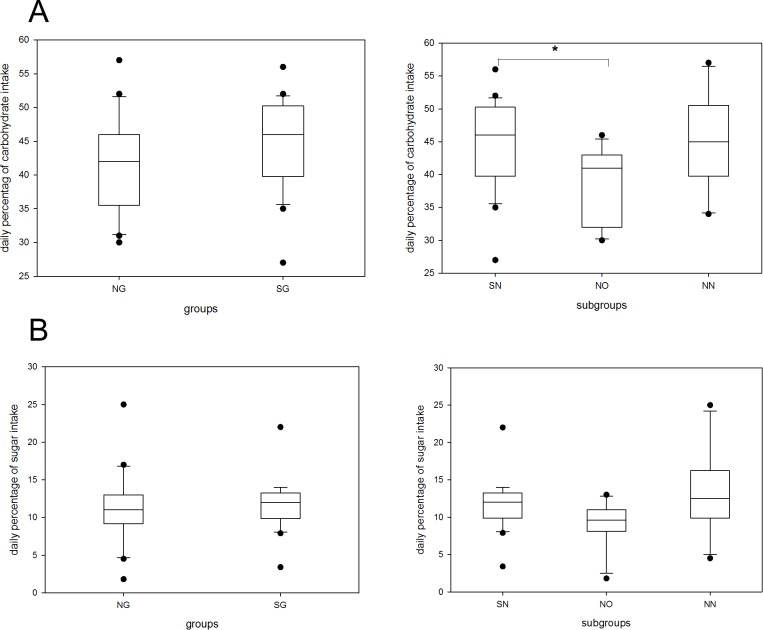
**Daily carbohydrate (A) and sugar (B) intake by groups and subgroups.** Carbohydrate (A) and sugar intake (B) as illustrated in percent. The boxes cover the first quartile on the bottom and the third quartile on the top. Whiskers reach from the minimum to the maximum value excluding outliers (illustrated by dots). Shift-working group and shift-working nursing-staff subgroup cover identical cohorts. NG, non-shift-working group; SG, shift-working group; SN, shift-working nursing-staff subgroup; NO, non-shift-working office-staff subgroup; NN, non-shift-working nursing-staff subgroup *p<0.05.

Analysis of fat consumption revealed a higher percental intake among the office staff (NO) and this difference was significantly different from both the shift working and non-shift working nurses (p<0.01) (Fig 7B). On the other hand, percental carbohydrate intake was less in the office group compared to shift working nurses (Fig 8A). The percental intake of sugar, representing partially snacking, did not withstand Bonferroni correction (using two tailed t-values) but showed a tendency towards less consumption in the NO subgroup (Fig 8B).

## Discussion

In a microepidemiological approach this study aimed to identify differences of lifestyle habits in individuals employed in health care in a German tertiary medical centre and working on either a shift or a non-shift schedule. We included parameters for physical and metabolic activity, stress perception, sleeping quality and nutritional habits. Groups differed in the way, that shift workers were younger and more often smokers than non-shift workers. These differences are closely in line with the data from the Nurses’ Health Study (NHS) II, where higher smoking behaviour rates were found in earlier and midlife among rotating shift workers [29]. In regard to age, our findings support the NHS findings that an older nursing personnel tends to work in clinics rather than on wards.

So the discrepancy in age might be also explained by the fact that older nursing staff changes from shift to a regular work schedule. Notably, women were proportionally overrepresented in the non-shift-working group in our data.

We were able to show the variation of physical activity in nurses on a shift rotation compared to daytime only workers, which appeared significant during working hours. Moreover, they were partly more prone to mental stress, and significant differences were seen in the composition of meals.

### Physical activity and metabolism

The very limited sample of similarly designed studies compared mainly working schedule-associated activity parameters: A Turkish study with day and night-shift working nurses using the SWA found average MET-values of a mean of 1.99 (SD = 0.35) for day shifting and a mean of 1.83 (SD = 0.28) for ordinary service nurses during work [30]. This result is in line with our findings with shift working nurses somewhat exceeding that value (Fig 2B).

However, our study’s findings vary from another study proposing less sedentary behaviour and more light activity for rotating shift workers [13]. A homogenous overall activity profile in the investigated population could explain that difference.

We were surprised by the discrepancy between step-rate and overall physical activity (in METs). Step-rate was quantified using the accelerometer method and is frequently used for assessment of physical activity in clinical studies. Conceivably, pedometry based on the accelerometer probably is not as accurate as that of classical pedometers. Yet, there are studies that demonstrated similarities between these two methods in young healthy [31] as well as elderly individuals [32]. Discrepancies are seen in obese people [33]. Since the participants of our study were not obese we don’t assume a marked deviation of the accelerometry results. Our data suggest that the main activity during working hours derives from walking activities as we noticed a good correlation of individual METs and number of steps while working. On the other hand, physical activity during off hours may include “non-walking” activities. Household and social activities seem to be more relevant to physical activities than stepping.

Only a minority of all subjects achieved the recommended number of steps (Fig 3) of at least 10.000 per day for healthy adults (i.e. 6.94 steps·minute-1). This threshold value has been recommended in the Australian “10.000 steps project” which used this value for a freely accessible community health program [34]. Mostly employees working on non-shift rotations (NG group) and office workers fell below the recommended number of steps.

The fact that nurses on a shift schedule usually have to walk more during their working hours, for control rounds or food and drug dispensation explains this difference. Furthermore, their activities such as washing and patient mobilisation are physically demanding and, at night, done in smaller teams.

REE was similar in shift or non-shift working individuals. Despite shift-work’s suggested influence on metabolism through circadian desynchronization [4,35] and changes in melatonin release [3], beholding melatonin’s impact on metabolism [36], the average range of shift worker’s in our study seems to be normo-metabolic.

A reduced metabolism (lower REE) was observed in the non-shift nursing subgroup that appeared to contradict our quite homogeneous study group. Considering that higher age reduces metabolism and REE, the higher age of non-shift working nurses can explain this difference (Table 1). Some studies attribute weight gain to reduced PA and non-adjusted energy intake in advancing age [37].

Additionally, obese individuals tend to deviate from REE-estimating equations [38] due to reduced muscular mass and less energy requirement of fat cells. Therefore the lower REE of the NN subgroup can also be inferred from their high median BMI and body fat composition, whereas the homogenous BCM in all groups could independently predict similar REE.

### Stress

Stress perception showed variations in our study and we noticed a higher social overload (e.g. “I must frequently care for the well-being of others.”) [39] in shift-working individuals. Nurses either working on a day time or rotating schedule reported higher social overload than office employees and confirms studies that compared nurses with students [40].

Interestingly, the lack of social recognition (e.g. “Although I do my best, my work is not appreciated.”) [39] was much more often reported by nurses than by office staff. It somewhat explains the current shortage of nurses and explains the recruitment problems of nursing schools in Germany.

### Nutrition

Conflicting data exist on nutritional habits of (shift)-working employees. Increased energy intake and insufficient intake of vitamins and dietary fibres were previously reported for shift workers [41]. Although we did not directly screen for food frequency and enhanced snacking among shift working employees, we found more in-between snacking of shift workers when we analysed their food diaries. In office employees we found a higher consumption of fat but less carbohydrate and sugar intake compared to shift working nurses. One reason may be that office workers have more opportunities to consume cooked square meals for lunch due to their regular time schedule. Shift working nurses are more dependent on cold snacks or fast food brought in by delivery service. In contrast, fast food often contains a lot of fat. The intake of fat was lower in shift-working nursing subgroup, whereas a high amount of fat is also found in cooked square meals depending from the way of preparation.

In addition, the shortage of guaranteed breaks increases the need for a quickly available energy source.

Macronutrient analysis of our whole study group showed an increased intake of fat (>30%) and sugar (>10%) but reduced amounts of carbohydrates (<50%) in their diet and is in line with the so called western pattern diet, which is suspected to predispose to obesity and cardiovascular disorders [42]. According to a recently published study rotating shifts are an independent risk factor for being overweight, independent of nutritional habits and physical activity [43]. We were unable to see significant differences in median BMI but here further studies are warranted.

The large variation in energy intake seen in the shift working group may indicate disturbed eating habits (e.g. overeating or erratic fluctuations) as a result of shift work. Moreover, data of self-reported food diary should be interpreted with caution as underreporting of absolute food intake or inaccuracies of estimated portion sizes can occur [44,45].

### Limitations

There are limitations of our study. We didn’t allow for blinding and stratification so an observer effect cannot be ruled out. Our participants might have practiced a healthier life style during the observation time than usually being aware that their physical activity is recorded.

For the same reason the self-reported food diary can be manipulated by participants resulting in inaccurate information on nutritional habits. Although, physical activity varied widely, particularly among the SN subgroup.

Moreover part time workers were included as well. Nevertheless PA-parameters used in the study were balanced for time of wearing and independent from time-dependent influences.

Another limitation is the small simple size. This fact can weaken the power of the study’s claims. Possible confounding cannot be ruled out. Appropriate statistical tests were used to correct for this limitation. The small sample size might derive from limited voluntary participation and lacking rewards. Another obstacle for participation is due to the practibility of the armband itself (e.g. armband’s weight, interference in daily routine) and time-consuming recording nutrition in a food diary. However, the majority of available studies covering this topic have comparable sample sizes.

Some parameters like BMI and WHR are not easily comparable between different ages and gender. Nevertheless, we found a representative study group covering a wide range of participants.

The equalisation of moderate to vigorous physical activity (MVPA) with percental time of overall METs of ≥ 3.0 also contains certain limitations but has been widely used [46]. SWA has limitations and is less sensitive when metabolic activity of very light physical work, such as office work is recorded [21,47]. Physical activity may therefore have been underestimated for office workers (NO subgroup) in our study.

General statistic considerations as well as the study design do not admit the interpretation that the study’s findings in the different examined categories deliver causality. This study has a comparative approach and was not intended to measure health outcomes. Considering that shift work is associated with metabolic health risks and a lack of moderate PA with metabolic diseases, we did not find evidence that shift work increases PA in a manner overcome the increased health risk. Other factors may affect the health of shift workers and shift work appears to be an independent risk factor by itself.

## Conclusions

Health behaviour assessed by physical activity showed comparable results for shift- and non-shift (daytime) workers that partly contradict previous reports. Differences were found when volunteer’s people were at work. Most probably office workers compensate for the lack of physical activity during business hours in their free time.

To our knowledge this is the first comparative approach to shift and non-shift workers employed in the health care industry with regard to their physical activity, nutrition, metabolism, chronic stress and sleep. These findings and range of parameters have not been described in a central European cohort yet.

Shift-working had no overall effect on physical activity but was associated with different eating habits and greater stress levels. Office workers appear to compensate for less physical activity during work in their off hours.

Further studies should enrol a larger sample size and longer periods of monitoring, e.g. four weeks. Advanced developments like smaller and lighter fitness-tracking armbands could be used as actigraphy in future studies to encourage study participation. The relation between shift work and impairment should always be noted by comparing shift-working and non-shift-working study groups of similar working environment (e.g. health-care workers).

The amount of MVPA and overall activity measured by METs should be used in future studies due to their clinical relevance and reproducibility.

Considering the theoretical foundation of this study—the biopsychosocial model- further studies should also acquire data about e.g. individual education, social status, income etc. By obtaining a larger and more representative sample size, evaluation of shift-work’s retrospective impact on social parameters such as family structure, career could be realized.

## Supporting Information

S1 File Minimal data set. The minimal data set contains all the relevant underlying raw data processed in this manuscript.
(XLSX)

## Abbreviations

BCM: body cell mass
BIA: bioelectrical impedance analysis
BMI: body mass index
ECM: extracellular mass
MET: metabolic equivalent of task
MVPA: moderate to vigorous physical activity
NG: non-shift-working
NN: non-shift-working nursing subgroup
NO: non-shift-working office worker subgroup
PA: physical activity
PSQI: Pittsburgh-Sleeping-Quality-Index
REE: resting energy expenditure
SE: standard error
SG: shift-working group
SN: shift-working nursing subgroup
SWA: SensewearPro3 armband
WHR: waist-to-hip-ratio
